# Unexpectedly detected appendiceal perforation during endoscopic direct appendicitis therapy despite negative preoperative computed tomography imaging

**DOI:** 10.1055/a-2462-2098

**Published:** 2024-11-22

**Authors:** Jun Cai, Yanli Wang, Silin Huang, Suhuan Liao, Jianzhen Ren, Yitian Guo, Nan Liu

**Affiliations:** 1 701237 Department of Gastroenterology, South China Hospital, Medical School, Shenzhen University, Shenzhen, China; 2 701237 Department of Pediatrics, South China Hospital, Medical School, Shenzhen University, Shenzhen, China; 3 701237 Institute of Environment and Health, South China Hospital, Medical School, Shenzhen University, Shenzhen, China

Endoscopic direct therapy, such as endoscopic direct appendicitis therapy (EDAT) and endoscopic direct diverticulitis therapy (EDDT), are now the preferred treatments for acute uncomplicated appendicitis and diverticulitis 1 2 . EDAT stands out for its minimal invasiveness, facilitating real-time observation and targeted treatment, while also delivering high definition imaging that enhances diagnostic accuracy 3 . We report a case of appendiceal perforation that was adeptly diagnosed through direct visualization using a 9-Fr cholangioscope (EyeMax; Micro-Tech, Nanjing, China) ( Video 1 ).

An appendiceal perforation is unexpectedly detected during endoscopic direct appendicitis therapy despite there having been no evidence of this on preoperative computed tomography.Video 1

A 6-year-old girl presented with lower right quadrant abdominal pain, and a computed tomography (CT) confirmed the diagnosis of acute obstructive appendicitis, but did not initially indicate any signs of perforation ( Fig. 1 ). She was subsequently admitted for EDAT to alleviate her condition. During the procedure, the appendiceal orifice was found to be excessively inflamed ( Fig. 2 **a** ). Upon seamless insertion of the cholangioscope into the appendiceal lumen, purulent secretions were observed ( Fig. 2 **b** ), and a perforation was visualized ( Fig. 2 **c** ). The EDAT was promptly aborted, and the patient was swiftly transferred to undergo a laparoscopic appendectomy, which verified the presence of acute appendicitis with appendiceal perforation ( Fig. 3 ). The patient has since made an excellent postoperative recovery and has not experienced any subsequent discomfort.

**Fig. 1:**
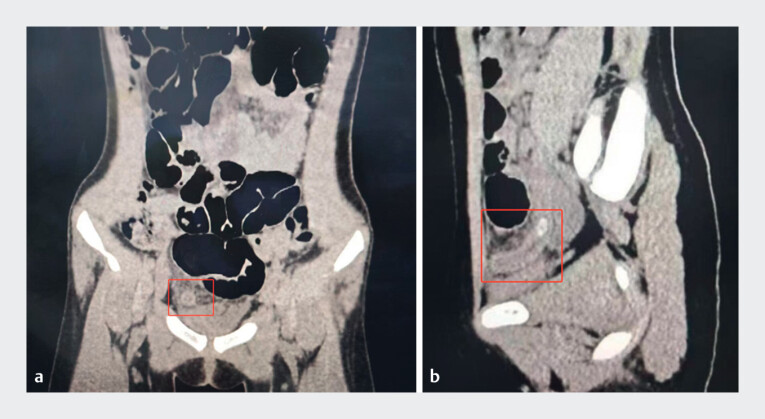
Computed tomography scan images showing: **a** on coronal view, an enlarged appendix (12 mm in diameter), edge-blurred, with no evidence of fluid or gas accumulation; **b** on sagittal view, significant thickening of the appendix, high density shadows in the lumen, and no evidence of fluid or gas accumulation.

**Fig. 2:**
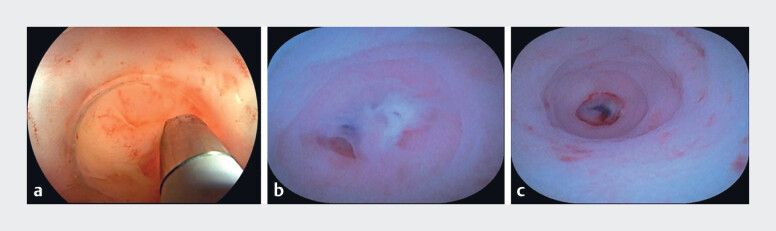
Images during endoscopic direct appendicitis therapy (EDAT) showing: **a** the cholangioscope passing into the appendiceal lumen; **b, c** on cholangioscopic view: **b** congestion, edema, and a small amount of purulent discharge in the appendiceal cavity; **c** an appendicular perforation.

**Fig. 3:**
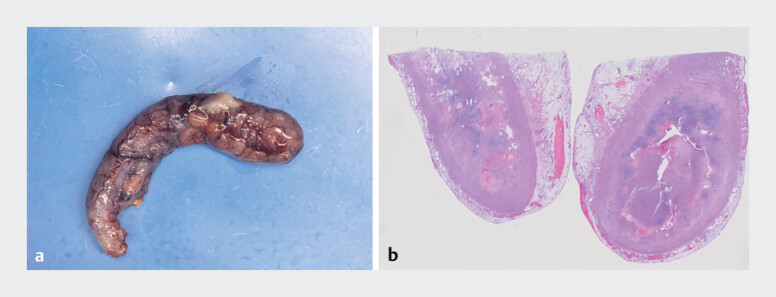
The postoperative specimen, which confirmed the appendiceal perforation, on: **a** macroscopic appearance; **b** histopathologic view, with obvious inflammation, edema, and a large amount of necrotic tissue in the cavity.

Typically, appendiceal perforation is diagnosed through a combination of clinical symptoms, abdominal ultrasound, and especially CT imaging 4 , necessitating immediate surgical intervention upon confirmation 5 . In this unique case, the CT scan failed to detect any signs of perforation; however, direct visualization with the cholangioscope did uncover the issue. This timely diagnosis facilitated immediate surgical intervention, thereby averting the escalation to more severe complications. To our knowledge, this represents the first case where appendiceal perforation was diagnosed via direct visualization cholangioscopy, providing an invaluable contribution to the rapid detection of such complications in the management of acute appendicitis.

Endoscopy_UCTN_Code_TTT_1AQ_2AG

## Notes

Endoscopy E-Videos https://eref.thieme.de/e-videos
*E-Videos* is an open access online section of the journal *Endoscopy* , reporting on interesting cases and new techniques in gastroenterological endoscopy. All papers include a high-quality video and are published with a Creative Commons CC-BY license. Endoscopy E-Videos qualify for HINARI discounts and waivers and eligibility is automatically checked during the submission process. We grant 100% waivers to articles whose corresponding authors are based in Group A countries and 50% waivers to those who are based in Group B countries as classified by Research4Life (see: https://www.research4life.org/access/eligibility/ ). This section has its own submission website at https://mc.manuscriptcentral.com/e-videos .
